# Supplementary figures and images for: Inositol trisphosphate and ryanodine receptor signaling distinctly regulate neurite pathfinding in response to engineered micropatterned surfaces
Source: PLoS One. 2024 Sep 5;19(9):e0308389. doi: 10.1371/journal.pone.0308389 (PMC11376539; doi:10.1371/journal.pone.0308389)

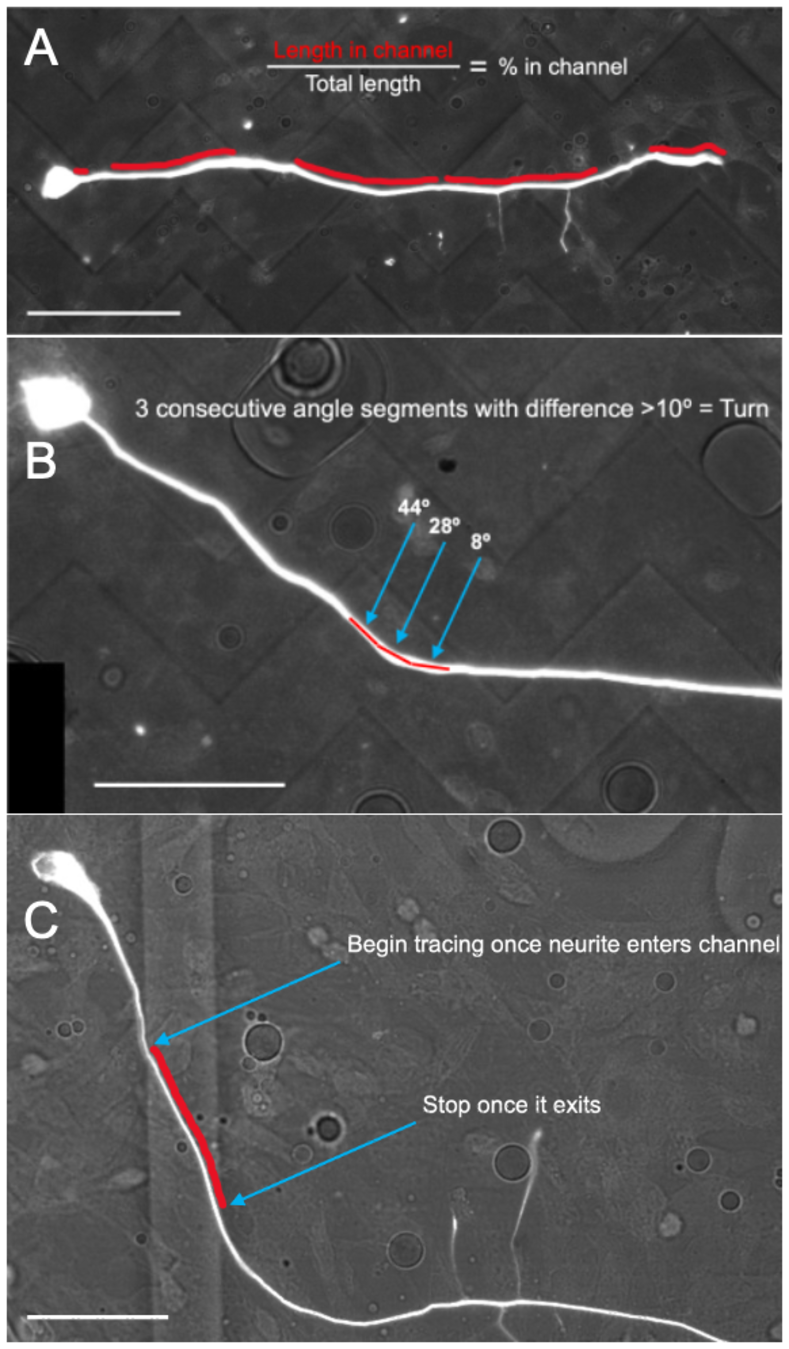

Supplement: S1 Fig — (A) Schematic demonstrating the derivation of percent of neurite length in topographical microfeature. (B) Schematic demonstrating calculation of turns. Neurite is segmented in to 10 μm segments and the angle relative to the horizontal calculated for each segment. The MATLAB program counts a turn when 3 consecutive segments vary by >10°. (C) Schematic demonstrating the measurement of the length a neurite remains in topographical microfeature. Scale bars = 50 μm. (TIF) [file pone.0308389.s001.tif]

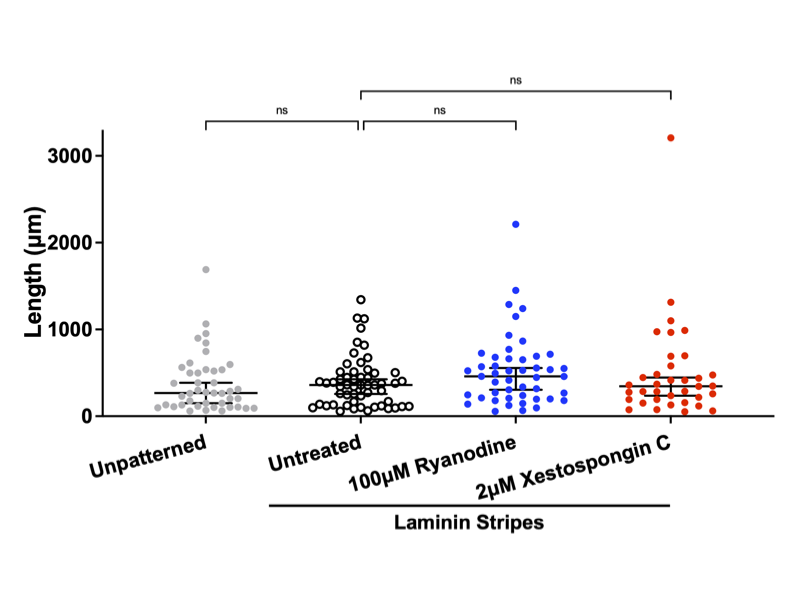

Supplement: S2 Fig — Total neurite length of SGNs cultured in the respective conditions. Kruskal-Wallis testing shows no effect of treatment on SGN neurite growth. Graph shows median Alignment Index +/- 95% CI. n = 38, 59, 52, 36 neurons. (TIF) [file pone.0308389.s002.tif]

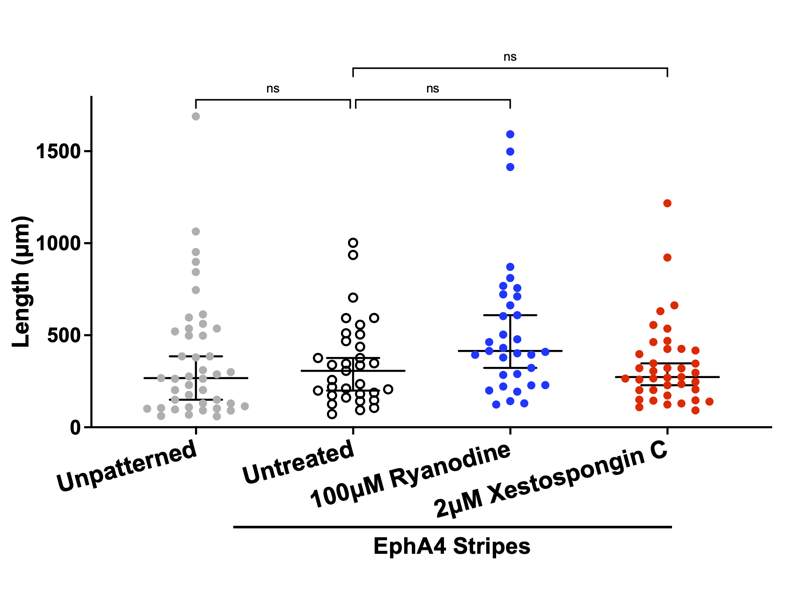

Supplement: S3 Fig — Total neurite length of SGNs cultured in the respective conditions. Kruskal-Wallis testing shows no effect of treatment on SGN neurite growth. Graph shows median Alignment Index +/- 95% CI. n = 38, 33, 33, 39 neurons. (TIF) [file pone.0308389.s003.tif]

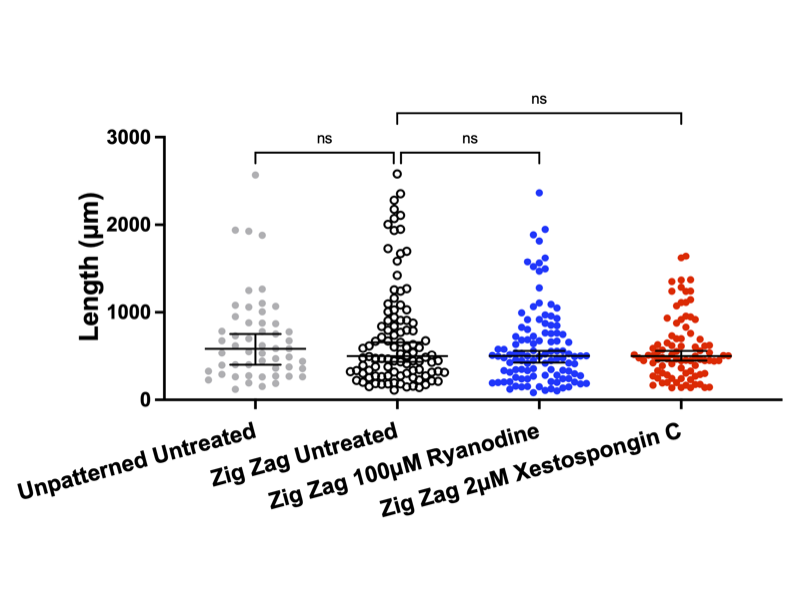

Supplement: S4 Fig — Total neurite length of SGNs cultured in the respective conditions. Kruskal-Wallis testing shows no effect of treatment on SGN neurite growth. Graph shows median Alignment Index +/- 95% CI. n = 53, 100, 109, 89 neurons. (TIF) [file pone.0308389.s004.tif]

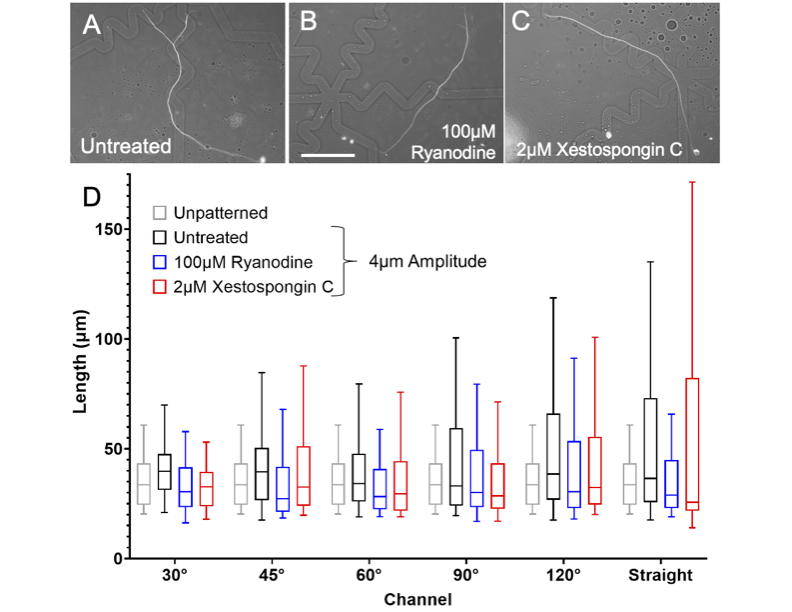

Supplement: S5 Fig — (A-C). Representative images of fixed SGNs growing in response to topographical microfeature turn challenges either untreated (A), treated with 100μM ryanodine (B), or 2μM xestospongin C (C). (D) Length neurite remains in topographical microfeature once encountering it. Two-way ANOVA on ranks suggests that both treatments impair the ability of SGNs to follow the topographical microfeature turn challenges and length followed increases with more gradual turns. Data are 95% confidence interval box and whisker. n = 29, 645, 355, 348 neurite encounters. Scale bar = 100 μm. (TIF) [file pone.0308389.s005.tif]
