# Supplementary material for: Inositol trisphosphate and ryanodine receptor signaling distinctly regulate neurite pathfinding in response to engineered micropatterned surfaces
Source: PLoS One. 2024 Sep 5;19(9):e0308389. doi: 10.1371/journal.pone.0308389 (PMC11376539; doi:10.1371/journal.pone.0308389)
Supplement: S1 File — (PDF) [file pone.0308389.s007.pdf]

Additional Description of Statistics and Data Visualization

Figure 1: Data are from two sets of independent variables (substrate and treatment) and the dependent variable has a normal distribution; thus, two-way ANOVA was used to compare data initially (see table below). Then follow up analysis was done with Dunnett's multiple comparison testing to compare individual treatment groups. Additionally, individual data points and SEM was to plot data since the sample size and distribution were relatively modest.

ANOVA table	SS (Type III)	DF	MS	F (DFn, DFd)	P value
Interaction	0.06834	2	0.03417	F (2, 176) = 2.389	P=0.0947
Drug	0.06392	2	0.03196	F (2, 176) = 2.234	P=0.1101
Substrate	0.1013	1	0.1013	F (1, 176) = 7.083	P=0.0085
Residual	2.517	176	0.01430		

Figure 2: D. Data are normally distributed thus SEM was used to highlight distribution and one-way ANOVA with follow-up Dunnett's multiple comparisons testing was done to compare groups. E. Data are not normally distributed, thus median +/- 95% confidence interval was used to highlight distribution and Kruskal-Wallis test with follow-up Dunn's multiple comparisons testing was done compare the groups.

Figure 3 and 4: Data are not normally distributed, thus median +/- 95% confidence interval was used to highlight distribution and Kruskal-Wallis test with follow-up Dunn's multiple comparisons testing was done compare the groups.

Figure 5: G. Data are proportions. Thus, standard error for a proportion was used to highlight uncertainty in the datasets and chi-square test with follow-up z-tests was done to compare groups. H & I. Data are normally distributed thus SEM was used to highlight distribution and one-way ANOVA with follow-up Dunnett's multiple comparisons was done to compare groups.

Figure 6: Data are from two sets of independent variables (channel and treatment) and the dependent variable does not have a normal distribution, thus two-way ANOVA on ranks was used (see table below). Additionally, due to the large data set with wide variation, box and whisker plot with 95% confidence was chosen to plot data.

ANOVA table	SS (Type III)	DF	MS	F (DFn, DFd)	P value
Interaction	26184	10	2618	F (10, 1330) = 0.6170	P=0.8004
Channel	187617	5	37523	F (5, 1330) = 8.842	P<0.0001
Drug	222417	2	111209	F (2, 1330) = 26.21	P<0.0001
Residual	5644206	1330	4244		

Figure 7: D. Data are from two sets of independent variables (channel and treatment) and the dependent variable is a proportion. Therefore, multinomial logistic regression was used to compare data (see table below). Additionally, all data for each treatment was compiled into overall and the proportions were compared with chi-square test with follow up z-tests to compare individual groups. E. Data are proportions. Thus, standard error for a proportion was

used to highlight the uncertainty in the datasets and chi-square test with follow-up z-tests was done to compare groups.

**Follow**	**Coefficient**	**SE**	**z**	**Lower 0.025**	**Upper 0.975**	**p-value**
**Ryanodine**	-0.7979	0.354	-2.256	-1.491	-0.105	0.024
**Xestospongin C**	0.1343	0.308	0.436	-0.47	0.739	0.663
**Turn Angle**	0.05708	0.0909	0.6278	-0.1211	0.235	0.530
**Align across zigzag**	**Coefficient**	**SE**	**z**	**Lower 0.025**	**Upper 0.975**	**p-value**
**Ryanodine**	-1.0887	0.443	-2.455	-1.958	-0.220	0.014
**Xestospongin C**	0.2570	0.350	0.734	-0.429	0.943	0.463
**Turn Angle**	-0.2483	0.107	-2.313	-0.03789	0.0207	-0.459
**Makes a turn**	**Coefficient**	**SE**	**z**	**Lower 0.025**	**Upper 0.975**	**p-value**
**Ryanodine**	-0.3268	0.566	-0.578	-1.435	0.782	0.563
**Xestospongin C**	-0.1999	0.568	-0.352	-1.313	0.914	0.725
**Turn Angle**	0.4631	0.188	2.460	0.8321	0.0139	0.0942

Figure 8: Data are from 3 independent variables (treatment) and the dependent variable is a classification into three groups/behaviors. Thus, chi-square test with z-tests was done to compare the treatment effect on the behaviors across groups.

Supplemental Figures 2-4: Data are from 4 treatment groups and the dependent variable is not normally distributed. Therefore, Kruskal-Wallis testing was conducted as a first step to compare the treatment groups.

Supplemental Figure 5: Data are from two sets of independent variables (channel and treatment) and the dependent variable does not have a normal distribution, thus two-way ANOVA on ranks was used (see table below). Additionally, due to the large data set with wide variation, box and whisker plot with 95% confidence was chosen to plot data.

ANOVA table	SS (Type III)	DF	MS	F (DFn, DFd)	P value
Interaction	26184	10	2618	F (10, 1330) = 0.6170	P=0.8004
Channel	187617	5	37523	F (5, 1330) = 8.842	P<0.0001
Drug	222417	2	111209	F (2, 1330) = 26.21	P<0.0001
Residual	5644206	1330	4244		
