# Supplementary material for: Inositol trisphosphate and ryanodine receptor signaling distinctly regulate neurite pathfinding in response to engineered micropatterned surfaces
Source: PLoS One. 2024 Sep 5;19(9):e0308389. doi: 10.1371/journal.pone.0308389 (PMC11376539; doi:10.1371/journal.pone.0308389)
Supplement: Attachment — Submitted filename: PLOS ONE Response.pdf [file pone.0308389.s008.pdf]

Dear. Dr. Sujeong Jang and the PLOS ONE editorial team,

We deeply appreciate the time and effort of the reviewers to critique our submission. A multi-disciplinary manuscript presents challenges to appropriately distill the background, motivations, and conclusions. The thoughtful comments and suggested revisions provided by the reviewers are immensely helpful. Outlined below are the responses to and the actions we took to improve the manuscript in response to each of the concerns brought up by the reviewers. These revisions significantly strengthen and clarify the manuscript and we hope that the revised manuscript is deemed suitable for publication in PLOS ONE. Thank you again for your consideration.

Response to Journal Requirements:

Comment 1: Please ensure that your manuscript meets PLOS ONE's style requirements, including those for file naming.

Response 1: The manuscript style was updated to reflect the formatting guidelines linked.

Comment 2: To comply with PLOS ONE submissions requirements, in your Methods section, please provide additional information regarding the experiments involving animals and ensure you have included details on (1) methods of sacrifice, (2) methods of anesthesia and/or analgesia, and (3) efforts to alleviate suffering.

Response 2: A sentence was added to the “Animals” section to clarify our use of animals.

Comment 3: Please note that funding information should not appear in any section or other areas of your manuscript. We will only publish funding information present in the Funding Statement section of the online submission form. Please remove any funding-related text from the manuscript.

Response 3: We have removed this from the “Acknowledgements” section

Comment 4: Thank you for stating the following financial disclosure: "MH and AG: NIH-NIDCD R01-DC012578, JV: NIH-NIDCD F31-DC020371, and University of Iowa: NIGMS T32-GM007337" Please state what role the funders took in the study. If the funders had no role, please state: "The funders had no role in study design, data collection and analysis, decision to publish, or preparation of the manuscript." If this statement is not correct you must amend it as needed. Please include this amended Role of Funder statement in your cover letter; we will change the online submission form on your behalf.

Response 4: We have added the statement: "The funders had no role in study design, data collection and analysis, decision to publish, or preparation of the manuscript." to the revised cover letter.

Comment 5: We note that your Data Availability Statement is currently as follows: "All relevant data are within the manuscript and its Supporting Information files."
Please confirm at this time whether or not your submission contains all raw data required to replicate the results of your study. Authors must share the “minimal data set” for their submission.

Response 5: We have added a raw data file which includes were used to generate Fig 5, Fig 6D, and S4 Fig. We have added these to accurately reflect the statement "All relevant data are within the manuscript and its Supporting Information files." and so that the submission now contains all raw data required to replicate the results of your study. The other data not directly addressed in this response are either plotted as scatter plots or are simple proportions. Please let us know if more raw data with respect to these are needed.

Comment 6: Please include your full ethics statement in the 'Methods' section of your manuscript file. In your statement, please include the full name of the IRB or ethics committee who approved or waived your study, as well as whether or not you obtained informed written or verbal consent. If consent was waived for your study, please include this information in your statement as well.

Response 6: More details of the ethics statement were added to "Animals" subsection of "Methods"

Comment 7: Please include captions for your Supporting Information files at the end of your manuscript, and update any in-text citations to match accordingly.

Response 7: The figure legends of the supplemental figures have been added to the end of the manuscript.

Response to Reviewer #1:

Comment 1: Results section – make findings clearer and more concise.

Response 1: We have reviewed and revised this section with focus on clarifying our interpretation of the results.

Comment 2: Line 21: do you need a – after Micro-? This recurs throughout the paper. Consider removing – on the first word. -This is repeated at other places in the paper

Response 2: We have removed the hyphen on this first usage and throughout the paper to clarify no hyphenation after "micro" or "nano"

Comment 3: Line 54: "into close proximity to stimulating" – difficult to read may want to change "to" to

Response 3: We have changed "into close proximity to stimulating" to "towards"

Comment 4: Successfully shows that disrupting Ca signaling alters neurite growth direction.

Response 4: We are glad that the reviewer agrees that our work demonstrates this.

Comment 5: Line 446: Is this 20% reduction significant?

Response 5: Yes both from a statistical and biological perspective. As stated in the figure legend for S4 Fig. "A Two-way ANOVA on ranks suggests that both treatments impair the ability of SGNs to follow the topographical microfeature turn challenges and length followed increases with more gradual turns." The methodology of this statistical analysis are found in the "Additional

Description of Statistics and Data Visualization" document as well. As noted in the comments to reviewer 2 below, we believe these differences carry significant biological relevance.

Comment 6: Good paper highlighting the importance in Ca pathways in direction of neurite growth. Excellent Figures.

Response 6: We appreciate the time and effort that the reviewer took to suggest revisions and critique our manuscript.

Response to Reviewer #2:

Comment: This study presents a comprehensive investigation into the role of IP3 and RyR signaling in neurite pathfinding of sensory neurons in response to biophysical cues in vitro. The manuscript is well-written, the conclusions are appropriate and supported by the data, and the findings are likely to be of interest to a wide audience as neurite growth is important for many biological processes and applications. The use of different combinations of micropatterned geometries and biochemical cues illustrates nicely the different functions of IP3 and RyR signaling in neurite pathfinding. The main drawback of this manuscript is that the effect sizes appear to be small and very specific to the measurements chosen, bringing into question the biological significance of the findings. Some clarification on the specific points below will help to address this concern.

Response: We appreciate the time and effort that the reviewer took to suggest revisions and critique our manuscript. A point-by-point response to each of their comments is below.

Comment 1: The introduction provides a good overview of the background into the field and existing technologies for the study of biochemical and biophysical cues for neurite outgrowth. The motivation for the study is generally clear although the authors could revise the paragraph on page 3 beginning on line 81 to more clearly state where the knowledge gap is within Ca2+ signaling and the RyR/IP3 pathways that this study addresses. Mainly, to answer the question of if is it known that they are all involved in neurite guidance, but it is unknown which type of environmental signal they respond to?

Response 1: We are glad that the reviewer believes that we have provided a good overview of the background. The reviewer is correct that there is a gap of knowledge in which environmental cues the Ca^{2+} signaling respond to. Additionally, another key point is that the "specific aspects (repulsion, turning, growth) of neurite pathfinding that these signaling pathways regulate" is also not well described. We have added more information to more clearly state the knowledge gap and what we attempted to address with this study.

Comment 2: Page 3, line 60: typo "In this, the growth cone plays a pivotal role in pathfinding responding to diverse…"

Response 2: We have added the word "by" into this sentence to correct the typo.

Comment 3: The authors should provide more rationale for the three micropatterned geometries chosen for this study. Some explanation is peppered through the results and discussion but clearer statements upfront would enhance the understanding of the findings

Response 3: Thank you for this helpful suggestion. We added sentences to the introduction to briefly explain the diversity of the substrates and our rationale for using them.

Comment 4: Since almost all experiments involve the use of ryanodine and xestospongin C the authors should include more explanation of how the concentrations were chosen and confirmed to inhibit RyR/IP3 signaling? Is inhibition with these drugs complete? Does administration of the drug affect cell viability?

Response 4: We have added this important clarifying information to the methods section and made a new subsection for this titled "Targeted inhibition of Ca^{2+} signaling".

Comment 5: In addition to the number of technical replicates (e.g., growth cones, etc), the biological sample size should be stated in the figure legends – i.e., the number of animals neurons were derived from. Can the authors also comment on how the statistical tests were conducted, i.e., which sample size was used for the analysis?

Response 5: We added clarifying information the methods "Spiral ganglion neuron cultures" section as well as to each figure legend where there was lack of clarity. Additionally, we refer the reviewer to the supplemental files where there is more description of our statistical methods since we know that we use diverse methods and some of the statistics are not straightforward.

Comment 6: Did the authors confirm that neuron viability is unaffected by culture on micropatterned substrates?

Response 6: We did not directly test neuron viability by the substrate pattern. Prior work has confirmed similar survival between SGNs grown on the polymer substrate and glass. We added a statement of this and added reference to the "Immunofluorescent labeling of SGNs" section of the methods. Importantly, all experiments took place on the same polymer substrates; even the unpatterned substrate is comprised of the same polymer. Importantly, we include neurotrophic factors in all culture media to support neuronal survival and neurite growth. Additionally, prior work confirms that the drug treatments do not affect SGN viability and we further show in the supplemental figures that neurite length is not impacted by the Ca^{2+} inhibitors.

Comment 7: Page 6, line 197: please revise sentence so it is clear which drugs are added to cells in these experiments

Response 7: See Response 5. We have added a section to the methods clarifying the use of drugs.

Comment 8: Page 6, line 201 and page 6, line 213: how were growth cones identified and ROIs selected? NF200 expression is not specific to the growth cone

Response 8: The real time imaging was conducted using rDRGNs cultured from mice expressing Pirt-GCaMP3. This fluorescent indicator protein fills the soma, neurite processes, and, importantly, the growth cone; the growth cones is visualized via epifluorescence microscopy. The ROI was a round shape approximated to best fill the growth cone without including extracellular area. This information was clarified in methods section.

Comment 9: Page 9, line 326: what is the biological relevance or significance of changes in growth cone morphology and orientation, and the relation to Ca2+ signaling?

Response 9: We discuss the biological relevance of these findings in the "IP3 and RyR act in the growth cone when neurites are navigating biophysical cues" section of the discussion. We have added a sentence to better describe the findings of Fig 2 in relation to Fig 1 in results section "Growth cone morphology on topographically patterned substrates is altered when inhibiting Ca^{2+} release from internal stores". This sentence should help highlight the implications of the findings of Fig 2 and its relation to Fig 1.

Comment 10: Since the analysis method appears to be unique to this manuscript and is somewhat complex to understand with text alone, Section 2.10, 2.11, and 2.12 may benefit from a supplementary figure illustrating the explanations of how neurite turning was classified and scored

Response 10: The methods in those sections are unique and are all methods that our research group has published previously. We have added reference to papers where we have previously generated figures describing these methods and cited them directly where the nuanced methods are mentioned. Additionally, we have created a supplemental figure to highlight novel methodologies, S1 Fig. These include: the MATLAB program calculating turns, percent length for a neuron in the micropatterned feature, and length of a neurite that remains in topographical microfeature once encountering it.

Comment 11: Page 10, line 369: The authors should provide references for the statement "EphA4-Fc serves as a chemorepulsive cue to SGN neurite growth…"

Response 11: We have added references for the line in question and better described that EphA4-Fc serves as a chemorepulsive cue to SGN neurite growth in the methods section.

Comment 12: The authors should consider also testing DRGNs in the more complex zigzag topographically patterned substrates to compare to the SGNs. It would be interesting to know if different types of sensory neurons responded differently to drug treatments

Response 12: This is a good idea for a next step to better study the ubiquity of these pathways across different types of sensory neurons. We chose to not explore this experiment for this initial study into sensory neurite guidance in response to diverse cues. Rather, we elected to study one neuron type for each experimental system to maximize our ability to explore diverse cues in one study. An important observation to note regarding this idea is that even though we do not compare the SGNs and DRGNs in the same experimental system with the drug treatments, the observations form the various systems agree. For example, the SGN findings from Fig 5 and 6 concur with the DRGN findings from Fig 7.

Comment 13: Can the authors comment on the effect of inhibiting RyR and IP3 together? Would there be an additive inhibitory effect on neurite growth? If those experiments were performed, please include in the manuscript, or if not authors can comment on the hypothesized effect

Response 13: This is another interesting idea for follow up work. We did not look at combined treatment group in this study since, as mentioned in Response 12, we prioritized creating numerous, diverse patterned substrates and measurements of growth for this study. We will hypothesize on the combined effect of both drugs in some detail here and add a brief comment to the manuscript summarizing this hypothesis and interest to pursue this in the future to the discussion.

In looking at the results from Figs 3, 4, and 5 we see a partial inhibition of neurite pathfinding when either the IP3 or RyR patherways is inhibited. Our hypothesized model in Fig 8 predicts a near total inability to be guided by these cues if both pathways are inhibited, as the growth would neither be able to stop nor turn in response to repulsive cues. Experiments to confirm this would include an analysis of alignment and morphology of neuronal cultures on various substrates with both pathways inhibited, comparing the measurements to those on an unpatterned substrate.
